# Neonatal Feeding Tube Colonization and the Potential Effect on Infant Health: A Review

**DOI:** 10.3389/fnut.2022.775014

**Published:** 2022-02-24

**Authors:** Leslie A. Parker, Marina Magalhães, Katelyn Desorcy-Scherer, Monica Torrez Lamberti, Graciela L. Lorca, Josef Neu

**Affiliations:** ^1^Department of Biobehavioral Nursing Science, College of Nursing, University of Florida, Gainesville, FL, United States; ^2^Department of Microbiology and Cell Science, Genetics Institute, Institute of Food and Agricultural Sciences, University of Florida, Gainesville, FL, United States; ^3^Division of Neonatology, Department of Pediatrics, College of Medicine, University of Florida, Gainesville, FL, United States

**Keywords:** infant, neonatal intensive care unit, feeding tube, contamination, colonization, enteral feeding, dwell time, infection risk

## Abstract

**Background:**

Infants in the neonatal intensive care unit (NICU) often require feeding tubes (FT) for weeks to months. Because FTs are in near constant contact with human milk and/or formula, rapid and extensive bacterial growth is possible. Due to their immature immunologic and gastrointestinal (GI) systems, infants may be at significant health risk due to FT colonization. In adults, length of time FTs remain in place (dwell time) affects the degree of colonization and biofilm formation which is important in infants whose tubes remain in place up to 30 days.

**Objective:**

The purpose of this review was to describe and summarize the evidence regarding FT bacterial colonization in infants and identify gaps needing further investigation.

**Methods:**

Medline, CINAHL, and Embase databases were searched for clinical and/or laboratory-based observational and randomized controlled studies investigating the presence of bacteria in neonatal FTs.

**Results:**

This review of 10 studies found evidence that neonatal FTs may contain high quantities of potentially pathogenic and antibiotic resistant bacteria and longer dwell times may increase the bacterial load. Furthermore, evidence suggests FT colonization may be nosocomial in origin and contribute to adverse infant health. Feeding tubes are an unrecognized source of bacterial colonization which may increase morbidity in premature infants and thus the presence of bacteria in FTs is an important area of investigation in the nutritional care of vulnerable infants in the NICU.

**Implications:**

Further appropriately powered studies which are clinically based, use appropriate analyses, and control for potential covariates are necessary to make clinical recommendations.

## Background

Due to prematurity and/or illness, infants in the neonatal intensive care unit (NICU) often require feeds to be provided via a feeding tube (FT). Feeding tubes are in near constant contact with formula or human milk providing ample nutrition for bacterial consumption and growth (1). Furthermore, frequent handling by health care workers may increase the risk of FT colonization which is especially important in infants in the NICU whose FTs are handled every 2–3 h in accordance with their feeding schedule (2). The presence of a FT is associated with an increased risk of late onset sepsis in preterm infants (3, 4) and it is possible this risk may be related to excessive FT colonization.

Previous research in adults indicates FTs are colonized with pathogenic bacteria and biofilm forms in the tube lumen (5). Biofilm formation is common in medical devices and significantly increases the risk of infection by enhancing bacterial resistance to antimicrobials and defending against environmental cleansing agents (6, 7). Because of their immature immunological and gastrointestinal (GI) systems, increased intestinal mucosal permeability, and often dysbiotic intestinal microflora, infants may be particularly vulnerable to FT colonization (2, 8, 9). Feedings administered through colonized FTs may introduce potentially pathogenic bacteria into the infant's GI system increasing the risk of adverse health outcomes. Provision of contaminated feedings are known to cause neonatal infection further suggesting that bacteria entering the infant's GI system through colonized FTs may be a risk for infection (2, 10).

The health risk associated with external devices such as central venous lines and urinary catheters is well-known to increase the longer the device remains in place (11). Because bacterial growth occurs rapidly in nutrient-rich environments, FT dwell time may influence bacterial growth. In adults, biofilm formation in FTs correlates with dwell time and develops in 60% of FTs in place <1 day, and 100% of tubes in place >1 day with dwell times >48 h resulting in dense biofilm formation (5, 12). In infants in the NICU, FTs often remain in place for up to 30 days likely increasing the risk for bacteria to multiply to potentially unsafe levels.

A systematic evaluation of available research on FT colonization in infants is lacking. Due to the potential health risk of colonized FTs in vulnerable infants in the NICU, the aim of this review is to describe and summarize the evidence regarding FT colonization in infants, to explore evidence on the association of FT dwell time and colonization and to demonstrate the need for further research.

## Methods

Clinical and laboratory studies investigating the presence of bacteria in infant FTs were included in this review. Clinical studies were limited to prospective observational studies and randomized control trials (RCTs).

### Search Strategy

To identify eligible studies, a search was conducted of the following electronic databases: Medline (through PubMed), CINAHL, and Embase. Additionally, the reference lists of included articles were examined. Our search was limited to studies published between January, 2000 to December, 2020 in English and used a combination of different groups of keywords [free text and MeSH (Medical Subject Headings) terms] that described the population and outcomes of interest (Supplementary Table 1). All titles and abstracts identified by our search were screened for relevance by a single author (LAP). The full texts of those considered relevant were retrieved and evaluated for inclusion relevancy by three authors (LAP, MM, and KD) who independently performed data extraction and collected the following information: first author's name, year of publication, study design, setting, and results.

**Figure 1:**
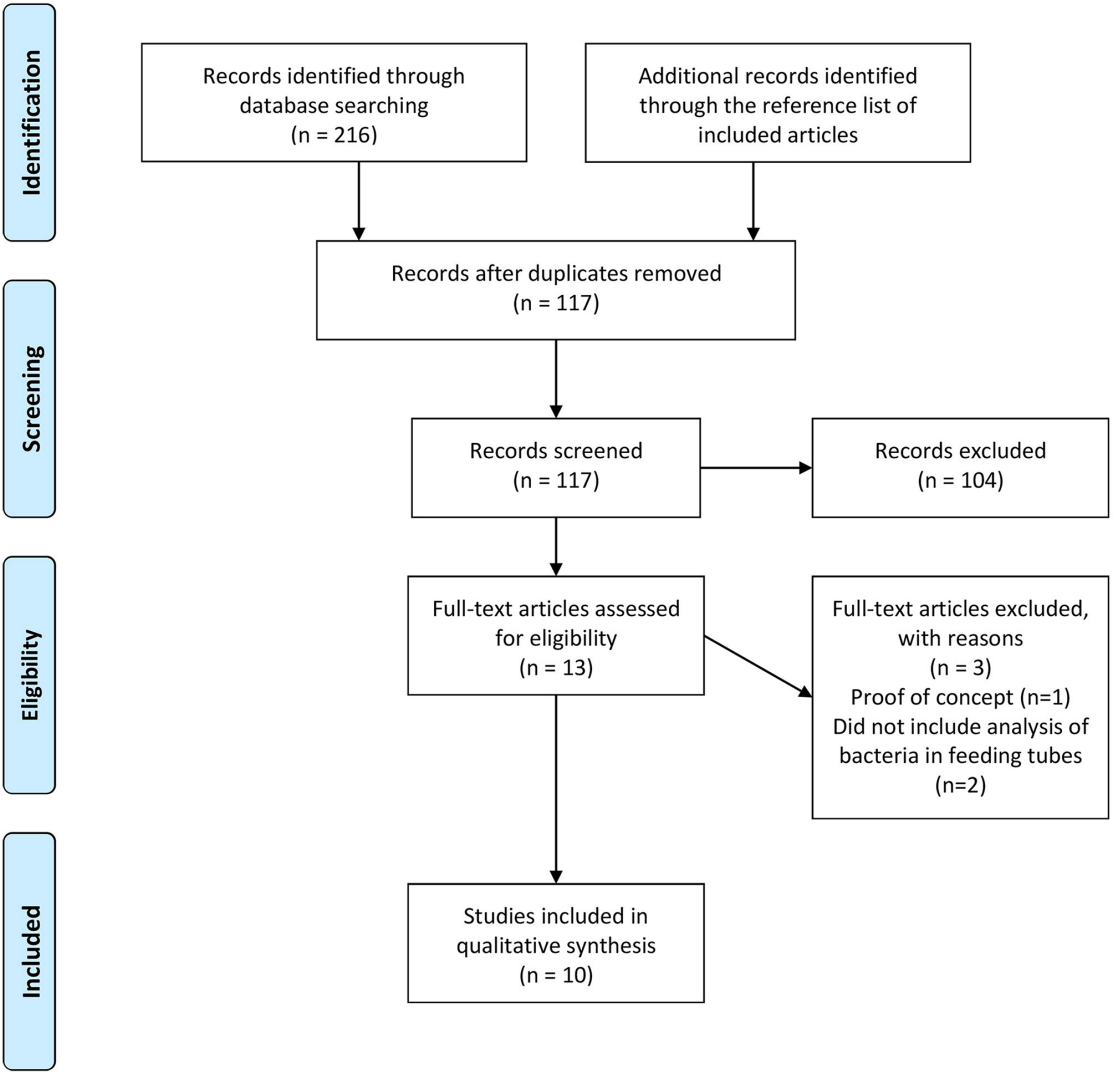
Flow diagram of search strategy and study selection.

A total of 216 articles were retrieved from the databases and reference lists of identified articles. After removing duplicates, 117 articles were screened by title and abstract. Of these, 104 were unrelated to FT colonization and excluded, leaving 13 articles potentially meeting inclusion criteria. After reviewing the full articles, 10 met criteria for inclusion in this review (Figure 1).

### Data Analysis

We synthesized data obtained from the included studies and all discrepancies between reviewers regarding study selection process, data extraction, synthesis, and interpretation were discussed until a final agreement was reached. In the following sections we summarize and discuss the findings of these studies and discuss future research priorities.

## Results

### Presence of Bacteria in FTs

All 10 included studies quantified or described the presence of bacteria in infant FTs (1, 2, 8, 9, 12–17). Five studies were laboratory-based which included studies that either analyzed inoculated FTs or performed additional analysis on specific bacteria isolated from FTs previously removed from infants in the NICU (1, 8, 13, 16, 17). Five were clinically based and included primary analysis of FTs removed from infants in the NICU (2, 9, 12, 14, 15). Two clinically-based studies included the same sample of infants and FTs (2, 13) and one laboratory-based studies (16) analyzed FTs obtained from clinically-based studies (9). Table 1 summarizes studies included for review.

**Table 1:** Summary of included studies.

**Study**	**Study design/Purpose**	**Setting and participants**	**Methodology**	**Results**
Mehall et al., 2002 (2)	Clinically-based. Prospective observational. Purpose: (1) to determine the incidence of feeding tube contamination and (2) to determine complications associated with feeding tube contamination.	Fifty infants with a mean gestational age of 28.5 weeks and mean birthweight of 1,387 g.	The lumen of 125 feeding tubes analyzed using standard culture techniques. Contamination was defined as >1,000 CFU/cm excluding *Staph epidermidis* and *alpha Streptococcus*. Culture results were compared with clinical cultures taken from infants in the NICU who were not enrolled in the study who were diagnosed with late onset sepsis using antibiotics susceptibility patterns and DNA electrophoresis.	Seventy-one (57%) of tubes were contaminated and only 8 (6%) were completely culture negative. The mean bacterial load was 908,173 CFU/cm. Feeding tubes from infants treated with histamine type 2 blockers were more likely to be contaminated (85.4 vs. 48.5%; *p* < 0.05). There was no association between antibiotic therapy and feeding tube contamination. Feeding intolerance only occurred in infants fed formula through contaminated tubes. In infants fed formula, feeding intolerance occurred more often when feeding tubes were contaminated (75 vs. 0%; *p* < 0.05). Ten infants had feeding tubes contaminated with >100,000 CFUs of Gram-negative rods (*Enterobacter or Klebsiella*) and seven of these infants developed necrotizing enterocolitis with four requiring surgery. Bacteria cultured from their peritoneal fluid was identical to the bacteria in their feeding tube. Infants gained more weight when fed through non-contaminated feeding tubes (178.9 vs. 94.2 g/week; *p* < 0.05). Feeding tube contamination was not associated with late onset sepsis. Organisms cultured from feeding tubes were identical to those subsequently causing infection both in infants who had the feeding tube and other infants in the NICU.
Mehall et al., 2002 (13)	Laboratory-based. Purpose: (1) To examine the presence of nosocomial antibiotic-resistant pathogenic bacteria in feeding tubes. (2) To determine whether bacteria in feedings tubes can cause infections in infants in the same NICU as infants with contaminated feeding tubes.	Hundred and twenty-five feeding tubes obtained from 50 infants previously analyzed by Mehall et al. (13).	Susceptibility to methicillin and vancomycin was determined on isolates of *Staphylococcus aureus* and *Enterococcus faecalis u*sing antibiotic susceptibility patterns and DNA pulse gel electrophoresis. Bacteria isolated from feeding tubes and from clinical cultures of infected infants were compared.	Twenty-three feeding tubes contained *S aureus* isolates, 12 of which were methicillin resistant. Four infants who did not have feeding tubes were diagnosed with MRSA infection in the NICU. DNA analysis indicated that the MRSA species was present in at least one of the cultured feeding tubes suggesting nosocomial transmission. There was no vancomycin resistant *Enterococcus*.
Kim et al., 2006 (1)	Laboratory-based. Purpose: To determine the effect of temperature and nutrients on biofilm formation by *Enterobacter sakazkii* in infant feeding tubes.	Number of feeding tubes analyzed was not reported.	Feeding tubes were inoculated with *Enterobacter sakazkii* and then immersed in either infant formula, tryptic soy broth, or lettuce juice broth and incubated at 12 or 25°C. The number of bacteria found in biofilm was determined for 10 days.	*Enterobacter sakazkii* attached to feeding tubes and as the biofilm aged, the cells detached. Biofilm formation did not occur at 12°C and formed at 25°C only when immersed in formula in which it grew to 1.16–1.31 log CFU/cm^2^.
Presti and Snyder, 2013 (17)	Laboratory-based. Purpose: To examine bacterial colonization in different areas of feeding tubes from several feeding tube designs.	*N* = 45 feeding tubes including five types which differed based on design and composition.	Feeding tubes were inoculated with a 2:1 mixture of formula and infant saliva. The hub, cap, and distal end of each type of tube were analyzed at 3-, 24-, and 72-h following inoculation using standard culturing techniques. Electron microscopy was performed on the plunger and recessed caps. Colony forming units (CFUs) were standardized for maximum growth for time and feeding tube part.	The most overall bacterial growth was seen at 3 h. Bacterial growth was significantly different based upon the design of the feeding tube cap at 3 (*p* = 0.04) and 24 h (*p* = 0.001) but not at 72 h. No difference in bacterial growth based on design of the distal end of the feeding tube or whether tubes were made of polyurethane or silicone. Bacteria were present in the hub, cap, and distal portion at all-time points but amounts were not reported.
Hurrell et al., 2009 (8)	Laboratory-based study. Purpose: To determine adherence to and growth of *Enterobacteriaceae* in feeding tubes.	Number of feeding tubes analyzed was not reported.	After being flushed with formula inoculated with *Enterobacteriaceae*, feeding tubes were then flushed with sterile formula. The feeding tube was examined for the presence of biofilm and viable counts reported. Formula flushed through the tubes was examined for bacteria using standard culturing techniques.	After 24 h, biofilm formation by *Enterobacteriaceae* was 10^5^-10^6^ CFU/cm. Bacteria in the tube lumen grew to cell densities of 10^7^ CFU/cm within 8 h and 10^9^ CFU/cm within 24 h. Bacteria in formula flushed through the feeding tubes grew to 10^8^-10^9^ CFU/ml within 24 h.
Hurrell et al., 2009 (9)	Clinically-based. Prospective observational. Purpose: To determine whether feeding tubes are colonized by *Enterobacteriaceae* and whether colonization is influenced by feeding regime.	Hundred and twenty-nine feeding tubes from 30 infants <1 week to >4 weeks of age collected from two NICUs.	The presence of *Enterobacteriaceae* in feeding tubes and residual liquid was determined using both standard culture and molecular methods and their antibiograms determined.	*Enterobacteriaceae* was isolated in 76% of feeding tubes including 52% of tubes from infants fed mother's own milk (MOM); and 78–88% of tubes from infants receiving either a combination of MOM and formula or formula. Analysis of 104 tubes obtained from one NICU found feeding regime was significantly associated with *Enterobacteriaceae* colonization (*p* < 0.0001) with tubes from infants fed MOM or who were nil by mouth having the lowest bacterial counts. The most common isolates were *Enterobacter cancerogenus, Serratia marcescens, Enterobacter hormaechei, Escherichia coli*, and *Klebsiella pneumonia*. Residual fluid contained a peak bacterial count of 10^7^ CFU/ml and contained the same *Enterobacteriaceae* species as in the feeding tube. Chronological age associated with the number of bacteria with an increase seen after 2 weeks (*p* < 0.001). A dwell time <6 h was associated with lower bacterial counts (*p* < 0.001). Resistance to amoxicillin and third generation cephalosporins was found on antibiogram.
Alkeskas et al., 2015 (16)	Laboratory-based. Purpose: To determine the diversity of *Escherichia coli* strains in previously isolated residual liquid from feeding tubes.	Sample included 30 strains of *Escherichia coli* isolated from residual liquid and biofilm obtained from 129 feeding tubes from 30 infants from two NICUs. Samples were collected in a previous prospective study (Hurrell et al., 2009a).	Strains of *Escherichia coli* were genotyped using pulsed-field gel electrophoresis, and seven-loci multilocus sequence typing. Potential pathogenicity of 30 virulence factors was determined by PCR-based assays and genome analysis.	Same strains of *Escherichia coli* were isolated from the residual liquid and the biofilm in 66% of feeding tubes which clustered into five pulsotypes.
Petersen et al., 2016 (14)	Clinically-based. Prospective observational. Purpose: To determine whether changing feeding tubes more frequently decreases contamination.	Ninety-four feeding tubes from 34 infants with a median gestational age of 30.1 weeks and median birthweight of 1,083 g. Median age of infant at collection was 37 days (1–119).	Saline flushed through feeding tube was analyzed using standard culture techniques. Potentially pathogenic bacteria were defined as either Gram-negative rods or *Staphylococcus aureus*. Biofilm within the feeding tube was visualized using scanning electron microscopy.	Eighty-nine percent of samples contained >1,000 CFU/ml of bacteria; 55% contained either *Enterobacteriaceae* or *Staphylococcus aureus*. Mean bacterial load was 5.3 log_10_ CFU/ml and maximum was 9.4 log_10_ CFU/ml. Median dwell time was 3.25 days (8 h−14.2 days). Neither the presence of bacteria (median 3.4 days if present vs. 3.2 days if not; *p* = 0.18) or presence of potentially pathogenic bacteria was associated with dwell time (median 3.7 days compared to 3.2 days; *p* = 0.54). Chronologic age was correlated with the presence of potentially pathogenic bacteria (*p* = 0.036). No difference in colonization of
				bacteria (*p* = 0.33) or the presence of potentially pathogenic bacteria (*p* = 0.077) based on antibiotic exposure. Use of probiotics did not increase the risk of contamination. A dense biofilm was observed using scanning electron microscopy in the inner surface of the feeding tube.
Gomez et al., 2016 (12)	Clinically-based. Prospective observational. Purpose: To evaluate bacterial colonization in external feeding tube systems (connected to the infant's feeding tube).	135 samples from 26 infants ≤ 32 weeks or ≤ 1,200 g. Samples included the feeding that passed through the external feeding system before entering the feeding tube.	Culture-based techniques were used to analyze samples. Scanning electron microscopy was used to visualize the biofilm present in six feeding tubes, connectors, and external feeding tubes from six infants.	*Staphylococcus* was present in 93% of MOM samples compared to 37% of DHM and 11% of formula feedings. *Enterococcus* was the most common Gram-positive bacteria found in DHM (49%) and formula (27%). Significantly more *Enterococcus* (*p* = 0.004); *Staphylococcus* (*p* < 0.001); and *Serratia* (*p* = 0.05) was found in MOM samples compared to other feeding types. At a species level, *Staphylococcus epidermidis* was the most abundant organism in MOM, *Enterococcus faecalis* was the most abundant in DHM and formula. Observed dense bacterial biofilms in external feeding tubes, feeding tubes, and connectors. The biofilm was particularly complex when the dwell time was >48 h and none was present in feeding tube with dwell times <12 h.
Taft et al., 2019 (15)	Clinically-based. prospective observational. Purpose: Increase understanding of biofilm formation in infant feeding tubes.	Ninety-seven feeding tubes from 47 infants with a mean gestational age of 32.3 weeks and a mean birthweight of 1,965 g.	Used 16S rRNA sequencing to characterize the bacterial composition of biofilms in different parts of feeding tubes (gastric, esophageal, pharyngeal) and to characterize bacteria in residual fluid. Whole metagenomics sequencing used to characterize antibiotic resistant genes (ARGs) present in a subset of feeding tubes from six infants.	Alpha diversity increased with gestational age, day of life, tube dwell time, and human milk feedings. Eight phyla were detected including Actinobacteria, Bacteroidetes, Cyanobacteria, Firmicutes, Fusobacteria, Proteobacteria, Tenericutes, and Thermi. Four families had median relative abundance greater than zero including *Enterococcaceae, Streptococcaceae, Staphylococcaceae, and Enterobacteriaceae*. ARGs differed based upon feeding regime but not between tube sections.

Four clinically-based (2, 9, 12, 15) and three laboratory-based studies (1, 8, 17) described the presence of bacteria within the FT lumen. Hurrel et al. (9) prospectively analyzed 129 FTs for *Enterobacteriaceae* finding a maximum bacterial load of 10^7^ CFU/tube while Mehall et al. (2) found 57% of 125 FTs removed from 50 infants to be contaminated (defined as >1,000 CFU of bacteria excluding *Staphylococcus epidermidis* and alpha-hemolytic *Streptococcus*) with a mean bacterial load of 908,173 CFU. Finally, Gomez et al. found dense biofilm but did not quantify how many FTs contained biofilm or the actual bacterial load (12) and Taft et al. using 16S rRNA sequencing to characterize the bacterial composition of biofilm found 100% of FTs contained bacteria (15). The three laboratory-based studies provided similar results (1, 8, 17). Hurrel et al. flushed FTs with formula inoculated with *Enterobacteriaceae* and found biofilm formation of 10^5^-10^6^ CFU/cm and a bacterial load up to 10^9^ CFU/cm (8). Similarly, Kim et al., inoculated FTs with *Enterobacter Sakaszkii* with biofilm formation after 12 h but only when incubated at 25 vs. 12°C (1). Finally, following inoculation of FTs with a 2:1 mixture of formula and infant saliva, Presti et al. found bacterial growth over time but did not report information regarding bacterial load (17).

Two clinically and one laboratory-based study analyzed residual fluid within FTs (9, 15, 16). Taft et al. found bacteria in the residual fluid of all FTs using 16S rRNA sequencing (15). Hurrell et al. found a peak bacterial load of 10^7^ CFU/ml with similar *Enterobacteriaceae* species as found in the FT lumen (9). Additional laboratory analysis of the residual fluid found 30 similar strains of *Escherichia coli* in the residual fluid and biofilm within the tube lumen (16).

Three studies examined fluid flushed through FTs (8, 12, 14). Petersen et al. found 89% of normal saline samples flushed through 94 FTs removed from 34 infants in the NICU contained >1,000 CFU/ml of bacteria with a mean bacterial load of 5.3 log_10_ CFU/ml, a maximum bacterial load of 9.4 log_10_ CFU/ml, and 55% contained either *Enterobacteriaceae* or *Staphylococcus aureus* (14). Similarly, Hurrel et al. found formula flushed through *Enterobacteriaceae* inoculated FTs contained 10^8^-10^9^ CFU/ml of bacteria within 24 h (8). Finally, after leaving the external feeding system before entering the actual FT, mother's own milk (MOM), donor human milk (DHM), and formula were found to contain bacteria (12). Because DHM and formula are considered sterile, these findings suggest bacteria in the external system may penetrate feedings as they pass through.

### Identification of Bacteria in FTs

Five studies identified the type of bacteria present in analyzed samples (2, 9, 12, 15, 16). Mehall et al. found the most common bacterial species in FT lumens included *S. epidermidis, S. aureus, Enterococcus faecalis, Enterobacter cloacae*, and *Klebseilla* (2), while Hurrell et al. found the most common *Enterobacteriaceae* isolates included *Enterobacter cancerogenus, Seratia marcescens, Enterobacter hormaechei, E. coli*, and *Klebsiella pneumonia* (9). When pulsed-field gel electrophoresis and seven-loci multilocus sequence typing was used to genotype *E. coli* in these samples, they clustered into five main pulsotypes known to cause serious and potentially fatal neonatal infection (16). It is important to note that FTs from 11 individual infants contained identical strains of *E. coli* K1 suggesting a common source of this pathogenic strain of bacteria. Similarly, after passing through the external feeding system, *Staphylococcus* was present in 93% of MOM feedings, 37% of DHM, and 11% of formula feedings (12). Finally, 16s rRNA gene sequencing found the most common phyla in biofilm were Proteobacteria, Firmicutes, and Actinobacteria and the most common genera were *Staphylococcus, Enterococcus*, and *Enterobacteriaceae* (15).

### Antibiotic Resistant Bacteria

Antibiotic resistance was investigated in three studies (9, 13, 15). When susceptibility to methicillin and vancomycin on isolates of *S. aureus* and *E. faecalis* was performed on FTs from infants in the NICU, 12 of 23 *S. aureus* isolates were methicillin resistant but none of the 71 *Enterococcus* isolates were vancomycin resistant (13). Resistance was also found in *Enterobacteriaceae* isolates in an additional clinically-based study where all strains of *Serratia marcescens* were resistant to amoxicillin and amoxicillin-clavulanic acid, while *E. hormaechei* strains were resistant to ceftazidime (21%) and cefotaxime (23%), and four of 37 *E. coli* strains were resistant to ceftazidime and cefotaxime (9). Finally, using whole metagenome sequencing of six FTs, Taft et al. found a median of 9.5–11 transferable antimicrobial resistance genes (ARGs) depending on the section of tube analyzed (15).

### Association of Dwell Time With FT Contamination

Six studies, four clinically (9, 12, 14, 15) and two laboratory-based (8, 17), explored bacterial growth in FT over time. Following inoculation with *Enterobacteriaceae* and flushing with infant formula, Hurrell et al., found FTs contained up to 10^7^ CFU/cm of bacteria by 8 h and 10^8^-10^9^ CFU/cm by 24 h with biofilm formation measuring 10^5^-10^6^ CFU/cm after 24 h (8). The second laboratory study found the highest bacterial growth occurred over the first 3 h followed by 24 and 72 h after FTs were flushed with a mixture of formula and infant saliva, incubated for 72 h and then analyzed (17). The four clinically-based studies reported mixed results. Hurrell et al., found FTs with longer dwell times contained higher bacterial loads (*p* < 0.001) with a dwell time <6 h having the lowest bacterial counts compared to between 6 and >48 h (9). Similarly, analysis of six FTs found complex biofilm formation after 48 h dwell time and no biofilm in tubes with dwell times of <12 h (12). Increased Alpha diversity indicating an increased number of species was also observed in FTs with longer dwell times (15). In contrast, Petersen et al. reported no association between dwell time and the presence of bacteria or the presence of potentially pathogenic bacteria but comparisons were made between dwell time differences of <1 day which may be insufficient for differences in growth to occur (14).

### Infant Outcomes Related to FT Colonization

One clinically-based and one laboratory based study investigated the association of FT colonization and adverse health outcomes (2, 13). Mehall et al. found only infants who received formula rather than human milk experienced feeding intolerance, with a greater incidence when fed through contaminated FTs (*p* < 0.05) (2). Of the 50 infants enrolled in the study, 10 had FTs containing >100,000 CFU/ml of gram negative rods and of those, seven developed necrotizing enterocolitis (NEC) with four requiring surgery. When surgery was required, bacteria cultured from the peritoneal fluid were identical to those found in each individual infant's FT. Furthermore, infants fed through colonized FTs experienced less mean weekly weight gain (178.9 vs. 94.2 g; *p* < 0.05). While colonized FTs were not associated with an increased risk of late onset sepsis, when infants were diagnosed with late onset sepsis, bacteria cultured from FTs were identical to the causative bacteria. In addition, organisms cultured from FTs of infants enrolled in the study were identical to those in blood cultures obtained from other infants in the NICU suggesting nosocomial spread of infection causing organisms. DNA pulse gel analysis of bacteria obtained from these FTs found the same species of MRSA as those infecting four infants residing in the same NICU but not enrolled in the study further suggesting nosocomial spread (13).

### Potential Variables Affecting FT Colonization

Several studies included potential confounders for the presence of bacteria and bacterial growth in FTs including gestational and chronologic age, birthweight, and feeding regime, as well as exposure to histamine 2 (H2) blockers, probiotics, and antibiotics. Five studies (two including the same group of infants) reported gestational age and birthweight with mean gestational age and birthweight ranging from 27.2 to 32.3 weeks and 1,083 to 1,965 g, respectively (2, 12–15). However, only one included either as covariates finding greater microbial diversity in FTs from infants with more advanced gestational ages (15).

Three studies examined the effect of chronologic age on the presence of bacteria in FTs (9, 14, 15). Higher chronologic age was associated with greater amounts of *Enterobacteriaceae* (9), an increased risk of colonization with potentially pathogenic organisms (14), and higher microbial diversity (15). However, the GI microbiome changes significantly following birth and differences may simply reflect the developing microbiome (18).

Feeding regime was included in three studies (9, 12, 15). Hurrel et al., found type of feeding was associated with the amount of *Enterobacteriaceae* (*p* < 0.0001) with infants receiving exclusive MOM feedings or nil by mouth having FTs with the lowest bacterial counts (9). While bacteria were found in all types of feeds, after passing through the external feeding system, compared to DHM or formula, MOM contained more *Enterococcus* (*p* = 0.004), *Staphylococcus* (*p* < 0.001), and *Serratia* (*p* = 0.05) (12). Finally, higher microbial diversity was found in FTs from infants fed either MOM or DHM (15).

Two studies reported whether infants received H2 blockers (2, 15) which could alter the microbial content of FTs by decreasing gastric acidity. Mehall et al. (2) found FTs from infants who received H2 blockers had an increased likelihood of containing >1,000 CFU/cm of bacteria compared to FTs obtained from infants who did not receive H2 blockers (85.4 vs. 48.5%; *p* < 0.05) while Taft et al. found no differences in alpha diversity when infants received H2 blockers (15).

Two studies included information regarding probiotics (14, 15). Peterson et al. found no significant effect of probiotic use on the presence of bacteria in normal saline flushed through FTs (14) and while an additional study included information regarding whether or not infants received probiotics, they did not include probiotic exposure in their analysis (15).

Antibiotic exposure was addressed in three studies and found to not affect the presence of >1,000 CFU/cm of bacteria (2), the presence of any bacteria, whether potentially pathogenic bacteria (defined as either Gram-negative rods or *S. aureus*) was present in saline flushed through FTs (14), or microbial diversity (15). However, definitions of exposure differed among studies and included exposure after birth (15), during the week tubes were removed (2), and while the tube was in place (14).

## Discussion

In this review, we found evidence that neonatal FTs contain bacteria which are potentially pathogenic and antibiotic resistant. FT colonization may allow bacteria to enter the infant's GI tract, potentially increasing the risk of complications including sepsis and NEC. Although external devices such as central venous lines and urinary catheters are known to increase morbidity, with risk increasing with longer dwell times, little attention has been paid to FTs as a possible risk to neonatal health (11). While the etiology of FT colonization is unknown, possible sources include bacteria present on the hands of parents and health care workers or in the infant's nasal or pharyngeal cavities (19). Furthermore, gastric bacteria may enter the FT during gastric residual evaluation or with gastroesophageal reflux (20). Because gastric pH is elevated in premature infants, gastric acidity may not impede microbial growth, potentially increasing the likelihood of FT colonization (21). Therefore, FT colonization is an important area of investigation in the care of infants in the NICU which has received little attention and for which further study is indicated.

Although evidence is limited, longer dwell times may be associated with higher levels of bacterial colonization. However, the optimal dwell time in neonates is unknown and while dwell times <12 h may limit bacterial growth, they are not clinically feasible due to workload and infant discomfort. The presence of bacteria for longer periods of time in a rich environment could enhance development of pathogens and horizontal gene transference, maximizing infant risk. A well-designed randomized controlled trial comparing more clinically appropriate dwell times is necessary to elucidate how often to change FTs and the effect of dwell time on infant health.

Inconsistencies between studies made comparisons challenging and included limiting analysis to specific families and genera of bacteria, analyzing different FT elements, and variations in type of analysis. In addition, inclusion of potentially confounding variables including gestational and chronologic age, birth weight, feeding regime, and exposure to medications/supplements were not consistent across studies potentially affecting results. Because human milk contains antimicrobial components and MOM contains an abundant and diverse microbiome (22), feeding both DHM and MOM likely affects the microbial contents of FTs (23, 24). However, no study analyzed bacteria in the feeding prior to infusion. Because higher gastric pH promotes bacterial growth, it is possible that H2 blocker exposure increases FT colonization yet was included as a covariate in only one study (2). Probiotics contain live bacteria and are increasingly administered to infants in the NICU, yet only two studies included probiotic exposure (14, 15). Finally, antibiotic exposure was only included in three studies, all of which differed in exposure definition (2, 14, 15). Future research which includes potential confounding variables is needed to elucidate their potential effect on FT colonization.

Because contaminated feedings are known to cause sepsis, meningitis, and NEC and bacteria known to cause these complications have been found in neonatal FTs (1, 9, 25), colonized FTs may increase the risk of infection. Furthermore, the presence of FTs is a known risk factor for sepsis (26, 27), and previous reports of identical strains of bacteria in the GI tract, blood, and FT of infants with late onset sepsis suggests FT colonization may increase the risk of sepsis in infants (2, 16). Finally, results of the study by Mehall et al. suggest FT colonization with pathogenic bacteria may be associated with NEC, feeding intolerance, and poor growth and that bacteria present in FTs may spread nosocomially (2, 13). Further research is necessary to determine the potential effect of FT colonization on neonatal health including whether the degree of colonization or type of bacteria influences risk of complications.

## Limitations of Included Studies

Selected studies are limited by small sample sizes, analysis of different FT elements, and different analysis methods. Lack of control for confounding factors could also have affected results. Finally, laboratory-based studies may not be representative of clinical settings and can't be used to make clinical practice recommendations.

## Conclusion

While this review found evidence that FT colonization may occur in infants in the NICU, information regarding potential effects on infant health is limited. Furthermore, while the degree of colonization, amount of biofilm formation, and number of Gram-negative bacteria present in FTs may be proportional to dwell time, insufficient evidence exists to inform clinical practice. Adequately powered studies using appropriate methodology and which consider relevant confounding variables are needed to fully understand to what extent FTs in the NICU contain bacteria, what level of colonization is considered safe and acceptable, type of bacteria which may be harmful, as well as factors which may increase or reduce the number of bacteria in FTs.

## Author Contributions

LP, MM, KD-S, MT, GL, and JN designed research. LP, MM, and KD-S conducted research and wrote the paper. LP had primary responsibility for final content. All authors read and approved the final manuscript and agree to be accountable for the content of the work.

## Funding

This work was supported in part by the University of Florida Graduate School and the National Institute of Nursing Research of the National Institutes of Health under Award Number 5R01NR016964-03.

## Conflict of Interest

The authors declare that the research was conducted in the absence of any commercial or financial relationships that could be construed as a potential conflict of interest.

## Publisher's Note

All claims expressed in this article are solely those of the authors and do not necessarily represent those of their affiliated organizations, or those of the publisher, the editors and the reviewers. Any product that may be evaluated in this article, or claim that may be made by its manufacturer, is not guaranteed or endorsed by the publisher.

## References

[1] Kim H Ryu JH Beuchat LR. Attachment of and biofilm formation by *Enterobacter sakazakii* on stainless steel and enteral feeding tubes. Appl Environ Microbiol. (2006) 72:5846–56. 10.1128/AEM.00654-06 16957203 PMC1563662

[2] Mehall JR Kite CA Saltzman DA Wallett T Jackson RJ Smith SD. Prospective study of the incidence and complications of bacterial contamination of enteral feeding in neonates. J Pediatr Surg. (2002) 37:1177–82. 10.1053/jpsu.2002.34467 12149697

[3] Petdachai W. Ventilator-associated pneumonia in a newborn intensive care unit. Southeast Asian J Trop Med Public Health. (2004) 35:724–9. 15689095

[4] Brito DV Brito CS Resende DS. Moreira do OJ Abdallah VO Gontijo Filho PP. Nosocomial infections in a Brazilian neonatal intensive care unit: a 4-year surveillance study. Rev Soc Bras Med Trop. (2010) 43:633–7. 10.1590/S0037-86822010000600006 21181013

[5] Leibovitz A Baumoehl Y Steinberg D Segal R. Biodynamics of biofilm formation on nasogastric tubes in elderly patients. Isr Med Assoc J. (2005) 7:428–30. 16011056

[6] Mittal S Sharma M Chaudhary U. Biofilm and multidrug resistance in uropathogenic *Escherichia coli*. Pathog Glob Health. (2015) 109:26–9. 10.1179/2047773215Y.0000000001 25605466 PMC4445292

[7] Lee HW Koh YM Kim J Lee JC Lee YC Seol SY. Capacity of multidrug-resistant clinical isolates of *Acinetobacter baumannii* to form biofilm and adhere to epithelial cell surfaces. Clin Microbiol Infect. (2008) 14:49–54. 10.1111/j.1469-0691.2007.01842.x 18005176

[8] Hurrell E Kucerova E Loughlin M Caubilla-Barron J Forsythe SJ. Biofilm formation on enteral feeding tubes by *Cronobacter sakazakii, Salmonella serovars* and other Enterobacteriaceae. Int J Food Microbiol. (2009) 136:227–31. 10.1016/j.ijfoodmicro.2009.08.007 19720416

[9] Hurrell E Kucerova E Loughlin M Caubilla-Barron J Hilton A Armstrong R. Neonatal enteral feeding tubes as loci for colonisation by members of the Enterobacteriaceae. BMC Infect Dis. (2009) 9:146. 10.1186/1471-2334-9-146 19723318 PMC2749046

[10] World Health Organization. Safe Preparation, Storage and Handling of Powdered Infant Formula Guidelines. (2007). Available online at: https://www.who.int/foodsafety/publications/micro/pif_guidelines.pdf (accessed December 1, 2019).

[11] Njere I Islam S Parish D Kuna J Keshtgar AS. Outcome of peripherally inserted central venous catheters in surgical and medical neonates. J Pediatr Surg. (2011) 46:946–50. 10.1016/j.jpedsurg.2011.02.037 21616258

[12] Gomez M Moles L Melgar A Ureta N Bustos G Fernandez L. Early gut colonization of preterm infants: effect of enteral feeding tubes. J Pediatr Gastroenterol Nutr. (2016) 62:893–900. 10.1097/MPG.0000000000001104 26741949

[13] Mehall JR Kite CA Gilliam CH Jackson RJ Smith SD. Enteral feeding tubes are a reservoir for nosocomial antibiotic-resistant pathogens. J Pediatr Surg. (2002) 37:1011–2. 10.1053/jpsu.2002.33831 12077760

[14] Petersen SM Greisen G Krogfelt KA. Nasogastric feeding tubes from a neonatal department yield high concentrations of potentially pathogenic bacteria- even 1 d after insertion. Pediatr Res. (2016) 80:395–400. 10.1038/pr.2016.86 27064248

[15] Taft DH Salinero LK Vongbhavit K Kalanetra KM Masarweh C Yu A. Bacterial colonization and antimicrobial resistance genes in neonatal enteral feeding tubes. FEMS Microbiol Ecol. (2019). 95:fiz039. 10.1093/femsec/fiz039 30915455 PMC6449222

[16] Alkeskas A Ogrodzki P Saad M Masood N Rhoma NR Moore K. The molecular characterisation of *Escherichia coli* K1 isolated from neonatal nasogastric feeding tubes. BMC Infect Dis. (2015) 15:449. 10.1186/s12879-015-1210-7 26497222 PMC4620641

[17] Presti A Snyder R. Enteral feeding tube design and differential bacterial overgrowth: an *in vitro* comparison. Neonat Intens Care. (2013) 26:25–9.

[18] Korpela K Blakstad EW Moltu SJ Strømmen K Nakstad B Rønnestad AE. Intestinal microbiota development and gestational age in preterm neonates. Sci Rep. (2018) 8:2453. 10.1038/s41598-018-20827-x 29410448 PMC5802739

[19] Aly H Badawy M Tomerak RH El-Kholy AA Hamed AS. Tracheal colonization in preterm infants supported with nasal continuous positive airway pressure. Pediatr Int. (2012) 54:356–60. 10.1111/j.1442-200X.2012.03567.x 22300448

[20] Bajorek S Parker L Li N Winglee K Weaver M Johnson J. Initial microbial community of the neonatal stomach immediately after birth. Gut Microbes. (2019) 10:289–97. 10.1080/19490976.2018.1520578 30404568 PMC6546338

[21] Milisavljevic V Garg M Vuletic I Miller JF Kim L Cunningham TD. Prospective assessment of the gastroesophageal microbiome in VLBW neonates. BMC Pediatr. (2013) 13:49. 10.1186/1471-2431-13-49 23560555 PMC3623619

[22] Groer MW Morgan KH Louis-Jacques A Miller EM. A scoping review of research on the human milk microbiome. J Hum Lactat. (2020) 36:628–43. 10.1177/0890334420942768 32735471

[23] Nolan LS Parks OB Good M. A review of the immunomodulating components of maternal breast milk and protection against necrotizing Enterocolitis. Nutrients. (2019). 12:14. 10.3390/nu12010014 31861718 PMC7019368

[24] Andreas NJ Kampmann B Mehring Le-Doare K. Human breast milk: a review on its composition and bioactivity. Early Hum Dev. (2015) 91:629–35. 10.1016/j.earlhumdev.2015.08.013 26375355

[25] Holý O Forsythe S. Cronobacter spp. as emerging causes of healthcare-associated infection. J Hosp Infect. (2014) 86:169–77. 10.1016/j.jhin.2013.09.011 24332367

[26] Hornik CP Fort P Clark RH Watt K Benjamin DK Jr Smith PB. Early and late onset sepsis in very-low-birth-weight infants from a large group of neonatal intensive care units. Early Hum Dev. (2012) 88(Suppl 2):S69–74. 10.1016/S0378-3782(12)70019-1 22633519 PMC3513766

[27] Troger B Gopel W Faust K Muller T Jorch G Felderhoff-Muser U. Risk for late-onset blood-culture proven sepsis in very-low-birth weight infants born small for gestational age: a large multicenter study from the German Neonatal Network. Pediatr Infect Dis J. (2014) 33:238–43. 10.1097/INF.0000000000000031 24030351

