# Supplementary material for: Neonatal Feeding Tube Colonization and the Potential Effect on Infant Health: A Review
Source: Front Nutr. 2022 Feb 24;9:775014. doi: 10.3389/fnut.2022.775014 (PMC8908000; doi:10.3389/fnut.2022.775014)
Supplement: Supplementary file 1 [file Table_1.DOCX]

**Supplementary Table S1:**

**PubMed Search Strategy**

("Enteral Nutrition"[Mesh] OR "feeding tube"[Text Word] OR "NG tube"[Text Word] OR "nasogastric tube"[Text Word] OR "orogastric tube"[Text Word] OR "OG tube"[Text Word] OR “enteral tube”[Text Word])

AND

("Equipment Contamination"[Mesh] OR "Cross Infection"[Mesh] OR "hospital-acquired infection"[Text Word] OR "nosocomial infection"[Text Word] OR "bacterial infection"[Text Word] OR contamination[Text Word] OR "Catheter-Related Infections"[Mesh] OR "infection prevention"[Text Word] OR "infection control"[Text Word] OR biofilm[Text Word] OR microorganism[Text Word] OR microbial[Text Word] OR bacteria[Text Word])

AND

("Infant"[Mesh] OR infant*[Text Word] OR neonat*[Text Word] OR baby[Text Word] OR NICU[Text Word])

**CINAHL Search Strategy**

((MH "Enteral Nutrition") OR "feeding tube" OR "NG tube" OR "nasogastric tube" OR "orogastric tube" OR "OG tube" OR “enteral tube”)

AND

((MH "Equipment Contamination") OR (MH "Infection Control+") OR (MH "Catheter-Related Infections") OR "hospital-acquired infection" OR "nosocomial infection" OR "bacterial infection" OR contamination OR "Catheter-Related Infections" OR "infection prevention" OR "infection control" OR biofilm OR microorganism OR microbial OR bacteria)

AND

((MH "Infant+") OR (MH "Neonatal Intensive Care Nursing") OR (MH "Intensive Care Units, Neonatal") OR infant* OR neonat* OR baby OR NICU)

**Web of Science Search Strategy**

("Enteral Nutrition" OR "feeding tube" OR "NG tube" OR "nasogastric tube" OR "orogastric tube" OR "OG tube" OR “enteral tube”)

AND

("Cross Infection" OR "hospital-acquired infection" OR "nosocomial infection" OR "bacterial infection" OR contamination OR "Catheter-Related Infections" OR "infection prevention" OR "infection control" OR biofilm OR microorganism OR microbial OR bacteria)

AND

(infant* OR neonat* OR baby OR NICU)
